# Coexistence of *bla*_NDM–5_ and *tet*(X4) in international high-risk *Escherichia coli* clone ST648 of human origin in China

**DOI:** 10.3389/fmicb.2022.1031688

**Published:** 2022-11-10

**Authors:** Muhammad Shafiq, Mi Zeng, Budi Permana, Hazrat Bilal, Jinhu Huang, Fen Yao, Abdelazeem Mohamed Algammal, Xin Li, Yumeng Yuan, Xiaoyang Jiao

**Affiliations:** ^1^Department of Cell Biology and Genetics, Shantou University Medical College, Shantou, China; ^2^School of Chemistry and Molecular Biosciences, University of Queensland, Brisbane, QLD, Australia; ^3^Department of Dermatology, The Second Affiliated Hospital of Shantou University Medical College, Shantou, China; ^4^Ministry of Education (MOE) Joint International Research Laboratory of Animal Health and Food Safety, College of Veterinary Medicine, Nanjing Agricultural University, Nanjing, China; ^5^Department of Pharmacology, Shantou University Medical College, Shantou, China; ^6^Department of Bacteriology, Immunology, and Mycology, Faculty of Veterinary Medicine, Suez Canal University, Ismailia, Egypt

**Keywords:** antimicrobial resistance, *Escherichia coli*, *bla*_NDM–5_, *tet(*X4*)*, coexistence, superbugs, hybrid plasmids

## Abstract

The emergence of pathogens is conferring resistance to last-resort therapies such as tigecycline, colistin, and carbapenems, limiting the therapeutic options, and raising concerns about the emergence of new “superbugs.” This study reports the first incident of a *bla*_NDM–5_ and *tet*(X4) co-harboring *Escherichia coli* with resistance to carbapenem and tigecycline recovered as the causative agent of a urinary tract infection in a 94-year-old patient. The *E. coli* strain ECCL209 carries multiple resistance genes [i.e., *bla*_TEM–1*B*_, *bla*_NDM–5_, *bla*_*CMY–*2_, *aadA22, florR, erm*(B), *mph*(A), *erm*(42), *lnuG*, *qnrS1*, and *sul*2] and exhibits resistance to almost all clinically used antibiotics. MLST analysis found that the strain belongs to ST648, considered a worldwide high-risk pandemic clone. Moreover, multiple plasmid incompatibility types were detected, i.e., IncHI1A, IncHI1B, IncFII, IncFIA, IncFIB, IncQ1, Col, and IncX4. Genetic analysis revealed that *bla*_NDM–5_ and *tet*(X4) genes were localized on two hybrid plasmids with multiple replicons. Continuous monitoring studies are suggested to quantify the antimicrobial resistance and assess the dissemination of such superbugs into a human healthcare setting.

## Introduction

Antimicrobial resistance (AMR) has been an emerging and increasing threat to global health (World Health Organization [WHO], 2014; Su et al., 2017). A report from 2016 predicted that global fatalities from infectious diseases caused by AMR will rise from 0.7 to 10 million by 2050, with a vast estimated inaction cost of US$100 trillion between 2016 and 2050 (O’Neill, 2016). The antibiotic-resistant bacteria (ARB) of particular interest are multidrug-resistant (MDR), extensively drug-resistant (XDR), and pandrug-resistant (PDR) (Basak et al., 2016). These ARBs are called superbugs, and they can cause severe bacterial infections due to their acquired and intrinsic resistance mechanisms and render the efficacy of many existing antibiotics (Potter et al., 2016; Acolatse et al., 2022).

Of particular concern is AMR among Gram-negative bacterial species, especially the carbapenem-resistant *Escherichia coli* (CRE), which is the leading cause of urinary tract infections (UTIs) and is challenging to treat with last-resort carbapenem antibiotics. Carbapenems were developed to tackle bacteria producing extended-spectrum β-lactamases (ESBLs). However, Gram-negative bacteria have become resistant to this group of drugs by developing and/or acquiring *bla* genes encoding carbapenem hydrolyzing enzymes, named carbapenemases (Codjoe and Donkor, 2017; Nordmann and Poirel, 2019). Among the newly emerging carbapenemases, New Delhi Metallo-β-lactamase (NDM) is very important due to its widespread dissemination and allelic variations (Suay-García and Pérez-Gracia, 2021). The pathogens harboring these genes resist almost all β-lactam antibiotics (Wu et al., 2019). Tigecycline and colistin were relatively effective and used as the last-resort treatments to treat such infections caused by MDR and XDR bacteria (He et al., 2019). However, the recent discoveries of plasmid-mediated colistin resistance genes (*mcr-1* to *mcr-10*) and/or the tigecycline resistance genes *tet*(X1) to *tet*(X15) among *Enterobacteriaceae*, especially in CRE, predict a return to the pre-antibiotic era and pose a severe threat to public health (He et al., 2019; Hussein et al., 2021). Furthermore, the co-occurrence of *tet*(X4) and *mcr-1* as well as the combination of *tet*(X4) and *bla*_NDM–5_ genes in tigecycline- colistin- and carbapenem-resistant *E. coli* strains recovered from animals in China, posing a significant threat to public health, which requires urgent monitoring in terms of its prevalence (He et al., 2020; Sun et al., 2021; Lu et al., 2022).

To the best of our knowledge, herein, we identified the first case of XDR *E. coli* isolate co-harboring plasmid-mediated *bla*_NDM–5_ and *tet*(X4) genes from a clinical sample from a human patient.

## Materials and methods

### Sample collection and identification

During a routine surveillance project on AMR, an *E. coli* isolate ECCL209 was recovered from a 94-year-old man, admitted for >6 months in the respiratory and critical care department at Shantou Hospital, Guangdong Province, China. The patient was diagnosed with UTI. The *E. coli* strain ECCL209 was identified by automated mass spectrometry systems (VitekMS, bioMerieux, Marcy I’Etoile, France) and further confirmed by PCR utilizing the primers specific to the *uidA* gene as reported previously (Shafiq et al., 2019).

### Antimicrobial susceptibility testing

Antimicrobial susceptibility testing was accomplished by Vitek 2 COMPACT (bioMerieux, Marcy I’Etoile, France) with AST-N334 cards for the following antimicrobial agents: amikacin (AMK), cefoperazone/sulbactam (SCF), cefepime (FEP), cefoxitin (FOX), cefotaxime (CTX), ertapenem (ETP), imipenem (IMP), amoxicillin/clavulanic acid (AMC), cefuroxime (CXM), ceftriaxone (CRO), ceftazidime (CAZ), piperacillin/tazobactam (TZP), ticarcillin/clavulanic (TCC), ceftazidime-avibactam (CZA), ciprofloxacin (CIP), doxycycline (DOX), tigecycline (TIG), aztreonam (ATM), minocycline (MIC), tobramycin (TOB), trimethoprim/sulfamethoxazole (SXT), and colistin (COL). Antibiotic susceptibility for levofloxacin (LEV) was determined using Levofloxacin Susceptibility Test Paper (Thermo Scientific™ Oxoid™, Leicestershire, United Kingdom). Results for all antibiotics were interpreted following the standard of the Clinical and Laboratory Standard Institute (CLSI M100; 31st edition) guidelines, except imipenem, ertapenem, amoxicillin-clavulanic acid, and ceftazidime/avibactam for which the European Committee on Antimicrobial Susceptibility Testing (EUCAST) breakpoints were considered.^1^

### Detection of antibiotic resistance genes

Detection of common ESBL genes (i.e., *bla*_TEM_, *bla*_CTX–M_, and *bla*_SHV_), carbapenemases (*bla*_NDM_, *bla*_KPC_, *bla*_IMP_, *bla*_VIM_, and *bla*_OXA_), and tigecycline-resistant genes *tet* (X3 and X4) was performed using PCR to identify resistance genes. All the primers used in this study are summarized in Supplementary Table 1.

### Mating assay

Conjugation experiments were performed according to a previously described method (Shafiq et al., 2019). The donor strain [*bla*_NDM_ and *tet*(X4)-positive *E. coli*] was diluted to the 0.5 McFarland standard and mixed with rifampicin-resistant recipient strain (*E. coli C600*) at a ratio of 1:1, respectively, on the microporous membrane. After cultures were incubated at 37°C for 12–14 h, the mixtures were collected and streaked on freshly made Luria-Bertani (LB) agar plates containing tigecycline (2 mg/L), meropenem (2 mg/L), and rifampicin (300 mg/L). The presence of *bla*_NDM_ and *tet*(X4) in transconjugants was confirmed by PCR and corresponding resistance phenotyping. The number of positive transconjugants per recipient calculated the transfer frequency of conjugation.

### Whole-genome sequencing with Illumina and Nanopore

To determine the genomic background, the ECCL209 *E. coli* strain was subjected to whole-genome sequencing (WGS) on the Illumina Miseq and Oxford Nanopore MinION platforms. The total DNA of *E. coli* strain ECCL209 was collected from fresh overnight cultures using a DNA kit (QIAamp^®^ DNA Mini Kit, Germany) according to the manufacturer’s guidelines. The quality and quantity of extracted genomic DNA were measured and confirmed using a Nanodrop OD-1000 spectrophotometer (Thermo-Scientific^®^). DNA libraries were constructed using NEBNext^®^ UltraTM DNA Library Prep Kit for Illumina (NEB, USA) and sequenced using an Illumina MiSeq sequencer (Illumina, San Diego, CA, USA). For the Nanopore platform, a Rapid Barcoding Sequencing Kit was used to construct the libraries and sequenced with a mini device (MinION), as previously reported (Maestri et al., 2019). Guppy base-calling software version 2.2 was used to generate fast5 files harboring the 1D DNA sequence from fast5 files. The quality of raw data from paired-end sequencing was checked using FastQC (version 0.11.6). Fastp (version 0.23.2) (Chen et al., 2018) was performed for the quality filtering to remove the low-quality reads, adapters, and poly-G tails. *De novo* assembly was accomplished using SPAdes (version 3.15.3) and Flye (version 2.8.3) with default parameters.

### Assembly annotation and genetic analysis of *Escherichia coli* ECCL209

The assembled genomes were subjected to determine the resistome, virulome, MLST, serotype, mobile genetic elements (MGEs), and plasmidome using online search tools such as ResFinder 4.0; VirulenceFinder 2.0, MLST 2.0, SerotypeFinder 2.0, MobileElementFinder, and PlasmidFinder 2.0, at the Center for Genomic Epidemiology (CGE).^2^ Genome annotation and visualization were performed using Prokka (version 1.14.6) and Proksee.^3^ Plasmid replicons were identified using Abricate (version 1.0.1)^4^ from the assemblies. EasyFig (version 2.2.2) was used to compare and visualize the region of interest between similar sequences. The sequence similarity search was performed using BLAST against the NCBI nucleotide database. The significant hits were investigated, and related information, including the source organisms and hosts, was visualized along the BLAST result tree using ggtree version 3.4.

## Results and discussion

The *E. coli* strain exhibited resistance against 19 antimicrobial agents, an XDR phenotype (Magiorakos et al., 2012), including SCF, FEP, FOX, CTX, ETP, IMP, AMC, LEV, TZP, CXM, CRO, CAZ, TCC, CZA, CIP, DOX, TIG, ATM, and MIC, while susceptible to AMK, TOB, SXT, and COL. The susceptibility data are shown in Supplementary Table 2. The *E. coli* isolate ECCL209 was resistant to carbapenems and tigecycline and harbored *bla*_TEM_, *bla*_NDM_, and *tet*(X4) genes, amplified by PCR and subsequently confirmed by Sanger sequencing.

To determine the transmissibility of *bla*_NDM_ and *tet*(X4) genes, we performed conjugation experiments with a recipient *E. coli* strain *C600.* The outcomes of conjugation proved that the *bla*_NDM_ and *tet*(X4) genes in donor *E. coli* isolate ECCL209, with their corresponding resistance against imipenem and tigecycline, were successfully moved to the recipient strain *C600*, suggesting that *bla*_NDM_ and *tet*(X4) genes were located on conjugative plasmids. The cotransfer of *bla*_NDM_ and *tet*(X4) was at a frequency of (1.67 ± 0.2) × 10^–1^ to (3.12 ± 0.1) × 10^–3^ cells per recipient.

The main comprehensive results from the WGS analysis of Illumina and Nanopore are summarized in Table 1. The ECCL209 isolate was assigned as serotype O83:H42 using SerotypeFinder 2.0,^5^ which is an extraintestinal pathogenic *E. coli* (ExPEC) primarily found in samples from animals, indicating their possible transmission from animal to humans (Abreu-Salinas et al., 2020; Shafiq et al., 2021a,b, 2022). MLST analysis revealed that *E. coli* isolate ECCL209 in this study belonged to sequence type (ST648), which had been previously reported to carry *bla*_CTX–M–_, *bla*_CMY–2–_, *bla*_NDM–_, *bla*_OXA–48–_, and *mcr-1* encoding genes and caused a significant proportion of infections in humans (Hornsey et al., 2011; Poirel et al., 2018; Chowdhury et al., 2022). This clonal lineage has emerged as a pandemic high-risk clone, being globally reported in humans, animals, and the environment (Hornsey et al., 2011; Fernandes et al., 2018; Furlan et al., 2020; Chowdhury et al., 2022; Landolsi et al., 2022). To the best of our knowledge, this is the first report of ST648, with *bla*_NDM_ and *tet*(X4).

**TABLE 1:** Genomic characteristics of *Escherichia coli* ST648 strain isolated from human origin.

Characteristics of *E. coli* ST648	Illumina (MiSeq)	Oxford Nanopore (MinION)
Source	Human urine	Human urine
Genome size (bp)	5,334,251	5,411,927
Contigs	176	4
G + C Content (%)	50.2	50.3
tRNA	83	88
rRNA	5	22
No. of CDS	5303	5,423
Serotype	O83:H42	O83:H42
*fimH*-type	H58	H58
ST	648	648
Mobilome	*IS5, ISL3, IS630, IS3, IS121, IS21, ISEcp1, IS4*	*IS5, ISL3, IS6, IS91, ISEcp1, IS21, IS4, IS110, IS30, ISAs1, IS630*
Virulome	*iutA, terC, IpfA, SitA, yfcV, terC, hra, eiIA, traT, chuA, air, iucC*	*traT, iucC, sitA, iutA, terC, lpfA, eilA, yfcV, chuA, gad, air, hlyE, hra*
**Resistome**		
Aminoglycosides	*aadA22*	*aadA22*
β-lactams	*bla*_NDM–5_, *bla*_TEM–1B_, *bla*_CMY–2_	*bla*_NDM–5_, *bla*_TEM–1B_, *bla*_CMY–2_
Chloramphenicol	*florR*	*floR*
Macrolides	*ermB*, *mphA, erm*(42), *lnuG*	*erm(B), mph(A), erm(42), lnu(G)*
Quinolones	*qnrS1*	*qnrS1*
Sulfonamides	*sul*2	*sul2*
Tetracyclines	*tet*(X4), *tetM*	*tet(X4), tet(M)*
Plasmidome	IncHI1A, IncHI1B, IncFII, IncFIA, IncFIB, IncQ1, Col, IncX4	IncHI1A, IncHI1B, IncFII, IncFIA, IncFIB, IncQ1, IncX4
BioProject accession number	PRJNA850111	PRJNA850111

ST, sequence type; CDS, coding sequences.

Our resistome results confirmed aminoglycosides (*aadA22*), amphenicols (*floR*), β-lactams (*bla*_TEM–1B_, *bla*_NDM–5_, and *bla*_CMY–2_), sulfonamides (*sul*2), macrolides [*ermB*, *mphA, erm*(42), and *lnuG*], quinolones (*qnrS1*), and tetracycline-resistant genes [*tet*(X4) and *tetM*]. Moreover, we found chromosomal mutations in *parE* (p. S458A), *parC* (p. S801), and *gyrA* (p. S83L, p. D87N), which encodes high-level resistance to fluoroquinolones (Mohsin et al., 2019). Multiple plasmids were detected in the *E. coli* ECCL209 strain, including, IncHI1A, IncHI1B, IncFII, IncFIA, IncFIB, IncQ1, Col, and IncX4. Detection of multiple plasmid types reflects the strains’ severity because all these replicons identified have the ability of horizontal transfer and play a vital role in spreading AMR genes (Rodríguez-Beltrán et al., 2021).

Regarding virulence genes, the presence of *iutA* (ferric aerobactin receptor), *terC* (tellurium ion resistance protein), *IpfA* (long polar fimbriae), *traT* (outer membrane protein complements resistance), *air* (enteroaggregative immunoglobulin repeat protein), *sitA* (iron transport protein), *hra* (heat resistance agglutinin), *yfcV* (fimbrial protein), *iucC* (aerobactin synthetase), *eilA* (*Salmonella HilA* homolog), and *chuA* (outer membrane hemin receptor) were identified in *E. coli* strain ECCL209. These virulence genes could enhance bacterial pathogenicity, and a recent study also described their direct interaction with ARGs in terms of bacterial survivability, which need to be disclosed in future studies (Zhang et al., 2019).

To further understand the genetic contexts of *bla*_NDM–5_ and *tet*(X4), we carried out long-read sequencing of *E. coli* ECCL209 isolate with the Oxford Nanopore MinION platform to obtain complete genome sequences. This assembled genome had four contigs, with a total length of 5,411,927 bp and an average G + C content of 50.31%. Bioinformatic analysis revealed that isolate ECCL209 harbored a chromosome and three circular plasmids comprising pECCL209-tetX4-190-kb, pECCL209-blaNDM5-157-kb, and pECCL209-blaCMY2-36-kb.

**FIGURE 1:**
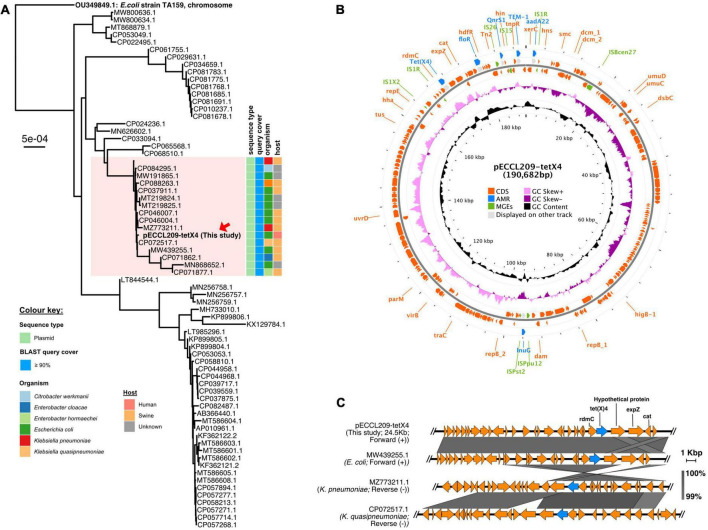
Structure of the *tet*(X4)-carrying plasmid and comparison of the genetic context of *tet*(X4). **(A)** BLAST tree comparison of plasmid pECCL209-tetX4 with other homologous plasmids available in the NCBI database. **(B)** Structure of the tet(X4)-carrying plasmid pECCL209. **(C)** Sequence comparison of the genetic context of a plasmid carrying *tet*(X4) gene from different sources. The arrows showed the direction of the transcription. Regions of >99% of homology are displayed by gray shading.

pECCL209-tetX4 was a 190,682-bp plasmid co-fused with IncHI1A, IncHI1B, and IncFIA, forming multiple replicon plasmids. Similarly, a fusion plasmid has been previously reported from China recently, where a *tet*(X4) gene was located in *Enterobacter cloacae* on a hybrid plasmid (∼190 kb) with IncFIA, IncHI1A, and IncHI1B replicons (Wu et al., 2022). This high homology of plasmids from animal and human origin suggests that *tet*(X4)-carrying plasmids could be conjugated from *E. cloacae* to *E. coli*. The BLASTn search was performed against the NCBI database to examine the sequence similarity of pECCL209-tetX4-190-kb and pECCL209-blaNDM5-157-kb. Phylogenetic analysis revealed that the pECCL209-tetX4 plasmid was similar to other bacterial strains with ≥90% query coverage. Most of the plasmid sequences matched with pECCL209-tetX4 were from animal origins, while this is the first human-origin *E. coli* plasmid harboring *tet*(X4) resistant gene (Figure 1A). The result showed that the pECCL209-tetX4-like plasmid might have been widely spread in different species of *Enterobacteriaceae*. pECCL209-tetX4-190-kb displayed a mosaic structure harboring five AMR genes, including *flor* (phenicol resistance), *qnrS*1 (quinolone resistance), *bla*_TEM–1B_ (β-lactam resistance), *aadA*22 (aminoglycosides resistance), and *lnu*(G) (lincosamide resistance), and the MGEs found in the MDR region were IS*26*, IS*Vsa3*, IS*6*, and IS*91* (Figure 1B). The backbone of plasmid pECCL209-tetX4-25-kb from this study harboring a *tet*(X4) gene showed >99% nucleotide homology and 100% query coverage to several other *tet*(X4) carrying plasmids in *Klebsiella pneumoniae* and *E. coli* reported from animal origins, including plasmid p3Z-5L-2-X4 (GeneBank accession no. CP072517.1) in *Klebsiella quasipneumoniae* and pTKPN_3-186k-tetX4 (GeneBank accession no. MZ773211.1) in *K. pneumoniae*, while plasmid pYUGZP1-tetX (GeneBank accession no. MW439255.1) in *E. coli* of unknown animal origin (Figure 1C). This high similarity and dissemination of this type of plasmid suggest that plasmids harboring *tet*(X4) had been widely propagated in animals (He et al., 2020; Sun et al., 2021; Lu et al., 2022). Moreover, the *tet*(X4) genetic context in plasmid pECCL209-tetX4 of this study showed a resemblance with the above three plasmids from the NCBI database, showing that the *tet(*X4) gene tends to be adjacent to the upstream *rdm*C gene and flanked by a complete IS*1R* element.

**FIGURE 2:**
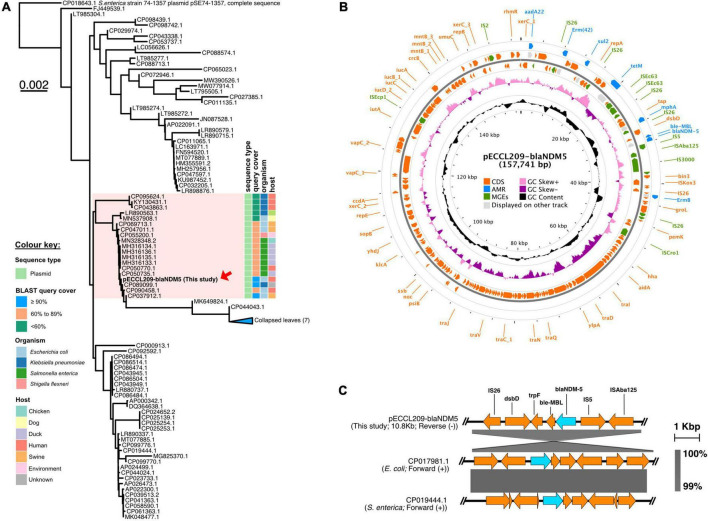
Structure of the *bla*_NDM–5_-carrying plasmid and comparison of the genetic context of *bla*_NDM–5_. **(A)** BLAST tree comparison of plasmid pECCL209-blaNDM5 with other homologous plasmids available in the NCBI database. **(B)** Structure of the *bla*_NDM–5_-carrying plasmid pECCL209. **(C)** Sequence comparison of the genetic context of a plasmid carrying *bla*_NDM–5_ gene from different sources. The arrows showed the direction of the transcription. Regions of >99% of homology are displayed by gray shading.

**FIGURE 3:**
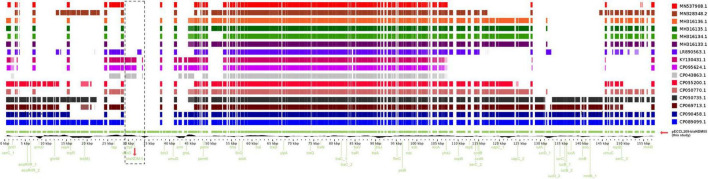
Linear alignment of the selected *bla*_NDM_ gene comparison with other homologous plasmids available in the NCBI database.

Similarly, pECCL209-blaNDM5 was a 157,741-bp hybrid plasmid with three replicon types, i.e., IncFII, IncFIA, and IncFIB. This plasmid showed ≥90% sequence identity with other *bla*_NDM–5_-carrying plasmids in *K. pneumoniae* plasmid pEH13_2 (GeneBank accession no. CP089099.1) and *E. coli* plasmid pYSP8-1-CTX-M-14 (GeneBank accession no. CP037912.1) of human and animal origin, respectively, suggesting that *bla*_NDM–5_-carrying plasmids had widely disseminated in China (Figure 2A). The *bla*_NDM–5_ gene resided in a complex region of the plasmid pECCL209-blaNDM5-157,741-bp. The plasmid carried other resistance genes, including *aadA22* (aminoglycosides resistance), *erm*(B), *erm*(42), *mph*(A) (macrolides resistance), *tet*(M) (tetracycline resistance), and *sul2* (sulfonamide resistance), and MGEs found in the MDR region, including IS*26*, IS*Vsa3*, IS*5*, IS*Ec9*, IS*Kox3*, and IS*91* (Figure 2B). The *bla*_NDM–5_ gene was located within a 10.8-kb region, which was highly similar (99% identity) to *E. coli* plasmid pGZ3_NDM5 (GeneBank accession no. CP017981.1) obtained from patient urine with intra-abdominal infections and *Salmonella enterica* plasmid unnamed2 (GeneBank accession no. CP019444.1) collected from a patient stool in China. In the plasmid backbone of pECCL209-blaNDM5-10kb, IS*5* was inserted with IS*Aba125* upstream of *bla*_NDM–5_, and the *ble*_MBL_ *trpF*, *dsbD*, and IS*26* were located downstream from *bla*_NDM–5_ as shown Figure 2C. Interestingly, on sequence alignment of our plasmid pECCL209-blaNDM5 with other identical plasmids ≥60% BLAST query coverage found that the *bla*_NDM–5_ region in other strains was mostly missing as shown in Figure 3, suggesting that *bla*_NDM–5_ in pECCL209-blaNDM5 was captured from other mobile elements.

## Conclusion

As far as we know, this is the first report that emphasized the emergence of high-risk *E. coli* clone ST648 of a human origin, which carries the mobile carbapenem and tigecycline resistance determinants *bla*_NDM–5_ and *tet*(X4), respectively. Regardless of their low prevalence rate in humans and animal-associated sources, the mobile plasmid-mediated resistance genes in such superbugs can pose a significant threat to public health. Therefore, continuous monitoring of such MDR and XDR bacteria in humans, animals, and the environment should be considered under the aegis of the One Health approach and to guide the deployment of public health interventions before clinical cases increase.

## Data availability statement

The sequence data mentioned in this present study were deposited to the GenBank NCBI database under the BioProject PRJNA850111 with accession number: SRR19844396.

## Ethics statement

Ethical approval was provided by the Human Research Ethics Committee of Shantou Central Hospital and Shantou University Medical College (Ref 047 and SUMC-2021-51, respectively). Consent forms from the patients were waived by the Ethical Committee as all the clinical samples were obtained from the hospital laboratory.

## Author contributions

MS and XJ designed the experiments. MS, MZ, and XL performed the experiments. MS wrote the original manuscript. BP and YY helped in the analysis. JH, HB, FY, and AA reviewed and edited the manuscript. All authors read and approved the final manuscript.

## Footnotes

1 http://www.eucast.org/clinical_breakpoints/, accessed on 30 July 2022.

2 http://www.genomicepidemiology.org/services/, accessed on 30 June 2022.

3
https://proksee.ca/

4
https://github.com/tseemann/abricate

5 http://www.genomicepidemiology.org/services/, accessed on 30 July 2022.

## Supplementary material

Supplementary Table 1 List of primers used in this study.

Supplementary Table 2 Antibiotic susceptibility profile of *Escherichia coli* strain ECCL209.

## References

[B1] Abreu-Salinas F. Díaz-Jiménez D. García-Meniño I. Lumbreras P. López-Beceiro A. M. Fidalgo L. E. (2020). High prevalence and diversity of cephalosporin-resistant *Enterobacteriaceae* including extraintestinal pathogenic *E. coli* CC648 lineage in rural and urban dogs in Northwest Spain. *Antibiotics* 9:468. 10.3390/antibiotics9080468 32752283 PMC7460362

[B2] Acolatse J. E. E. Portal E. A. Boostrom I. Akafity G. Dakroah M. P. Chalker V. J. (2022). Environmental surveillance of ESBL and carbapenemase-producing gram-negative bacteria in a Ghanaian Tertiary Hospital. *Antimicrob. Resist. Infect. Control* 11:49. 10.1186/s13756-022-01090-2 35296353 PMC8925048

[B3] Basak S. Singh P. Rajurkar M. (2016). Multidrug resistant and extensively drug resistant bacteria: A study. *J. Pathog.* 2016:4065603. 10.1155/2016/4065603 26942013 PMC4749793

[B4] Chen S. Zhou Y. Chen Y. Gu J. (2018). fastp: An ultra-fast all-in-one FASTQ preprocessor. *Bioinformatics* 34 i884–i890. 10.1093/bioinformatics/bty560 30423086 PMC6129281

[B5] Chowdhury G. Ramamurthy T. Das B. Ghosh D. Okamoto K. Miyoshi S. I. (2022). Characterization of NDM-5 carbapenemase-encoding gene (bla (NDM-5)) – positive multidrug resistant commensal *Escherichia coli* from diarrheal patients. *Infect. Drug Resist.* 15 3631–3642. 10.2147/IDR.S364526 35837541 PMC9275505

[B6] Codjoe F. S. Donkor E. S. (2017). Carbapenem resistance: A review. *Med. Sci.* 6:1. 10.3390/medsci6010001 29267233 PMC5872158

[B7] Fernandes M. R. Sellera F. P. Moura Q. Gaspar V. C. Cerdeira L. Lincopan N. (2018). International high-risk clonal lineages of CTX-M-producing *Escherichia coli* F-ST648 in free-roaming cats, South America. *Infect. Genet. Evol.* 66 48–51. 10.1016/j.meegid.2018.09.009 30227226

[B8] Furlan J. P. R. Savazzi E. A. Stehling E. G. (2020). Widespread high-risk clones of multidrug-resistant extended-spectrum β-lactamase-producing *Escherichia coli* B2-ST131 and F-ST648 in public aquatic environments. *Int. J. Antimicrob. Agents* 56:106040. 10.1016/j.ijantimicag.2020.106040 32479889

[B9] He T. Wang R. Liu D. Walsh T. R. Zhang R. Lv Y. (2019). Emergence of plasmid-mediated high-level tigecycline resistance genes in animals and humans. *Nat. Microbiol.* 4 1450–1456. 10.1038/s41564-019-0445-2 31133751

[B10] He T. Wei R. Li R. Zhang L. Sun L. Bao H. (2020). Co-existence of tet(X4) and mcr-1 in two porcine *Escherichia coli* isolates. *J. Antimicrob. Chemother.* 75 764–766. 10.1093/jac/dkz510 31840165

[B11] Hornsey M. Phee L. Wareham D. W. (2011). A novel variant, NDM-5, of the New Delhi metallo-β-lactamase in a multidrug-resistant *Escherichia coli* ST648 isolate recovered from a patient in the United Kingdom. *Antimicrob. Agents Chemother.* 55 5952–5954. 10.1128/AAC.05108-11 21930874 PMC3232805

[B12] Hussein N. H. Al-Kadmy I. Taha B. M. Hussein J. D. (2021). Mobilized colistin resistance (mcr) genes from 1 to 10: A comprehensive review. *Mol. Biol. Rep.* 48 2897–2907. 10.1007/s11033-021-06307-y 33839987

[B13] Landolsi S. Selmi R. Hadjadj L. Ben Haj Yahia A. Ben Romdhane K. Messadi L. (2022). First report of extended-spectrum β-lactamase (bla(CTX-M1)) and colistin resistance gene mcr-1 in *E. coli* of lineage ST648 from cockroaches in Tunisia. *Microbiol. Spectr.* 10:e0003621. 10.1128/spectrum.00036-21 35230131 PMC9045256

[B14] Lu X. Du Y. Peng K. Zhang W. Li J. Wang Z. (2022). Coexistence of tet(X4), mcr-1, and bla(NDM-5) in ST6775 *Escherichia coli* isolates of animal origin in China. *Microbiol. Spectr.* 10:e0019622. 10.1128/spectrum.00196-22 35311537 PMC9045152

[B15] Maestri S. Cosentino E. Paterno M. Freitag H. Garces J. M. Marcolungo L. (2019). A rapid and accurate MinION-based workflow for tracking species biodiversity in the field. *Genes* 10:468. 10.3390/genes10060468 31226847 PMC6627956

[B16] Magiorakos A.-P. Srinivasan A. Carey R. B. Carmeli Y. Falagas M. Giske C. (2012). Multidrug-resistant, extensively drug-resistant and pandrug-resistant bacteria: An international expert proposal for interim standard definitions for acquired resistance. *Clin. Microbiol. Infect.* 18 268–281. 10.1111/j.1469-0691.2011.03570.x 21793988

[B17] Mohsin M. Azam M. Ur Rahman S. Esposito F. Sellera F. P. Monte D. F. (2019). Genomic background of a colistin-resistant and highly virulent MCR-1-positive *Escherichia coli* ST6395 from a broiler chicken in Pakistan. *Pathog. Dis.* 77:ftz064. 10.1093/femspd/ftz064 31782775

[B18] Nordmann P. Poirel L. (2019). Epidemiology and diagnostics of carbapenem resistance in gram-negative bacteria. *Clin. Infect. Dis.* 69 S521–S528. 10.1093/cid/ciz824 31724045 PMC6853758

[B19] O’Neill J. (2016). *Tackling drug-resistant infections globally: Final report and recommendations*, ed. Ro A. (London: HM Government and Wellcome Trust).

[B20] Poirel L. Madec J. Y. Lupo A. Schink A. K. Kieffer N. Nordmann P. (2018). Antimicrobial resistance in *Escherichia coli*. *Microbiol. Spectr.* 6:14. 10.1128/microbiolspec.ARBA-0026-2017 30003866 PMC11633601

[B21] Potter R. F. D’souza A. W. Dantas G. (2016). The rapid spread of carbapenem-resistant *Enterobacteriaceae*. *Drug Resist. Updates* 29 30–46. 10.1016/j.drup.2016.09.002 27912842 PMC5140036

[B22] Rodríguez-Beltrán J. Delafuente J. Leon-Sampedro R. Maclean R. C. San Millan A. (2021). Beyond horizontal gene transfer: The role of plasmids in bacterial evolution. *Nat. Rev. Microbiol.* 19 347–359. 10.1038/s41579-020-00497-1 33469168

[B23] Shafiq M. Huang J. Rahman S. U. Shah J. M. Chen L. Gao Y. (2019). High incidence of multidrug-resistant *Escherichia coli* coharboring mcr-1 and blaCTX-M-15 recovered from pigs. *Infect. Drug Resist.* 12 2135–2149. 10.2147/IDR.S209473 31410033 PMC6643958

[B24] Shafiq M. Huang J. Shah J. M. Ali I. Rahman S. U. Wang L. (2021a). Characterization and resistant determinants linked to mobile elements of ESBL-producing and mcr-1-positive *Escherichia coli* recovered from the chicken origin. *Microb. Pathog.* 150:104722. 10.1016/j.micpath.2020.104722 33421607

[B25] Shafiq M. Huang J. Shah J. M. Wang X. Rahman S. Ali I. (2021b). Characterization and virulence factors distribution of bla CTX-M and mcr-1 carrying *Escherichia coli* isolates from bovine mastitis. *J. Appl. Microbiol.* 131 634–646. 10.1111/jam.14994 33411963

[B26] Shafiq M. Rahman S. U. Bilal H. Ullah A. Noman S. M. Zeng M. (2022). Incidence and molecular characterization of ESBL-producing and colistin-resistant *Escherichia coli* isolates recovered from healthy food-producing animals in Pakistan. *J. Appl. Microbiol.* 133 1169–1182. 10.1111/jam.15469 35094463

[B27] Su J.-Q. An X.-L. Li B. Chen Q.-L. Gillings M. R. Chen H. (2017). Metagenomics of urban sewage identifies an extensively shared antibiotic resistome in China. *Microbiome* 5:84. 10.1186/s40168-017-0298-y 28724443 PMC5517792

[B28] Suay-García B. Pérez-Gracia M. T. (2021). “Present and future of carbapenem-resistant *Enterobacteriaceae* infections,” in *Advances in clinical immunology, medical microbiology, COVID-19, and big data*, ed. Bawa R. (New York, NY: Jenny Stanford Publishing), 435–456.

[B29] Sun H. Zhai W. Fu Y. Li R. Du P. Bai L. (2021). Co-occurrence of plasmid-mediated resistance genes tet (X4) and bla NDM-5 in a multidrug-resistant *Escherichia coli* isolate recovered from chicken in China. *J. Glob. Antimicrob. Resist.* 24 415–417. 10.1016/j.jgar.2021.02.010 33621691

[B30] World Health Organization [WHO] (2014). *Antimicrobial resistance global report on surveillance: 2014 summary.* Geneva: World Health Organization.

[B31] Wu W. Feng Y. Tang G. Qiao F. Mcnally A. Zong Z. (2019). NDM metallo-β-lactamases and their bacterial producers in health care settings. *Clin. Microbiol. Rev.* 32 e00115–e00118. 10.1128/CMR.00115-18 30700432 PMC6431124

[B32] Wu Y. He R. Qin M. Yang Y. Chen J. Feng Y. (2022). Identification of plasmid-mediated tigecycline-resistant gene tet(X4) in *Enterobacter cloacae* from pigs in China. *Microbiol. Spectr.* 10:e0206421. 10.1128/spectrum.02064-21 35230154 PMC9045145

[B33] Zhang B. Sun C. Xia Y. Hu M. Wen X. (2019). Profiles of antibiotic resistance genes and virulence genes and their temporal interactions in the membrane bioreactor and oxidation ditch. *Environ. Int.* 131:104980. 10.1016/j.envint.2019.104980 31295641

