# Supplementary material for: Coexistence of blaNDM–5 and tet(X4) in international high-risk Escherichia coli clone ST648 of human origin in China
Source: Front Microbiol. 2022 Nov 10;13:1031688. doi: 10.3389/fmicb.2022.1031688 (PMC9685555; doi:10.3389/fmicb.2022.1031688)
Supplement: Supplementary Table 1 — List of primers used in this study. [file Table_1.DOCX]

| Primers | Sequence (5’ to 3’) | Amplicons size |
| --- | --- | --- |
| NDM-F  NDM-R | GGTTTGGCGATCTGGTTTTC  CGGAATGGCTCATCACGATC | 621 |
| OXA-F  OXA-R | GCTTGATCGCCCTCGATT  GATTTGCTCCGTGGCCGAAA | 281 |
| IMP-F  IMP-R | TTGACACTCCATTTACAG  GATCGAGAATTAAGCCACCC | 139 |
| VIM-F  VIM-R | GATGGTGTTTGGTCGCATA  CGAATGCGCAGCACCAG | 390 |
| KPC-F  KPC-R | CATTCAAGGGCTTTCTTGCTGC  ACGACGGCATAGTCATTTGC | 538 |
| CTX-F  CTX-R | CGC TTT GCG ATG TGC AG  ACC GCG ATA TCG TTG GT | 550 |
| TEM-F  TEM-R | CATTTCCGTGTCGCCCTTATTC  CGTTCATCCATAGTTGCCTGAC | 800 |
| SHV-F  SHV-R | AGCCGCTTGAGCAAATTAAAC  ATCCCGCAGATAAATCACCAC | 713 |
| tet(X3)- F  tet(X3)-R | CAGGACAGAAACAGCGTTGC  GCAGCATCGCCAATCATTGT | 179 |
| tet(X4)-F  tet(X4)-R | TTGGGACGAACGCTACAAAG  CATCAACCCGCTGTTTACGC | 181 |

**Table S1.** List of primers used in this study
